# Effect of Oat β-Glucan on the Rheological Characteristics and Microstructure of Set-Type Yogurt

**DOI:** 10.3390/molecules26164752

**Published:** 2021-08-06

**Authors:** Xiaoqing Qu, Yuliya Nazarenko, Wei Yang, Yuanyang Nie, Yongsheng Zhang, Bo Li

**Affiliations:** 1 School of Food Science, Henan Institute of Science and Technology, Xinxiang 453003, China; qingyang2719@163.com (X.Q.); ywcomwy@163.com (W.Y.); yuanyang8238@163.com (Y.N.); zlr1988@126.com (Y.Z.); 2 Department of Milk and Meat Technology, Sumy National Agrarian University, 40021 Sumy, Ukraine; nazarenko.sumy@gmail.com; 3 Henan Engineering Research Center of Fruit and Vegetable Processing and Quality Safety Control, Xinxiang 453003, China

**Keywords:** set-type yogurt, oat β-glucan, rheological characteristics, microstructure

## Abstract

The oat β-glucan (OG) was added into set-type yogurt as a functional ingredient, in order to evaluate effects on the rheological characteristics and microstructure of set-type yogurt. When the OG concentration increased from 0 to 0.3%, the WHC gradually increased. At 0.3% OG, the set-type yogurt had the highest WHC of 94.67%. Additionally, the WHC continuously decreased, reaching the lowest WHC (about 80%) at 0.5% OG. When 0.3% OG was added, the highest score of sensory evaluation was about 85. The rheological result showed that the fermentation process went through the changes as follows: solid → liquid → solid → liquid. The addition of 0.3% OG decreased the fermentation time of set-type yogurt by about 16 min, making yogurt more inclined to be liquid. The acidity of set-type yogurt with OG was slightly higher. The result of microstructure showed that the addition of OG destroyed the three-dimensional network structure of yogurt, and some spherical aggregate particles could be clearly observed at 0.3% OG. Overall, this study provided a theoretical basis for the application of OG in set-type yogurt.

## 1. Introduction

Because of the high nutritional value and health function, set-type yogurt has received increasing attention from consumers. The viscosity and the microstructure are important indexes to evaluate the quality of this type of product. The addition of thickener can improve the quality of yogurt. Previous research showed that cereal β-glucan can improve the structural characteristics of yogurt under the suitable concentration range, such as affecting the network structure of the protein, reducing the separation of whey, improving the water-holding ability and viscosity [1]. Oat β-glucan (OG) is a functional component that can reduce blood lipid, blood sugar, and enhance immunity [2,3]. Moreover, OG has better emulsifying stability and higher viscosity [4] and can be used as a food colloid and thickener in the food system. Brennan et al. added OG to low-fat yogurt, which significantly improved the viscosity of the product [5].

In addition, cereal β-glucan has been shown to have the property of prebiotic. In the gastrointestinal tract, cereal β-glucan acts as a prebiotic for microbial fermentation, selectively stimulating the growth and activity of some beneficial bacteria [6,7,8]. Shen et al. compared this with the prebiotic role of OG and barley β-glucan, who found that the mice intestines with OG were higher than that of barley β-glucan in the number of *Lactobacillus* and *Bifidobacterium*, which better promoted intestinal health [9]. Gee et al. studied the effects of barley β-glucan on the growth of two yogurt starter cultures composed of *Streptococcus thermophilus* and *Lactobacillus denderi*, subspecies *Bulgarian,* respectively, and who found that the barley β-glucan had no adverse effect on probiotic growth [10]. Ibrahim et al. studied the effects of OG and/or *bifidobacterium* producing exopolysaccharide on the physical properties, fermentation time, and sensory evaluation of low-fat yogurt, and the result showed that the OG and *bifidobacterium* produced exopolysaccharide in low-fat yogurt, improved the physical and sensory properties of yogurt, and enhanced probiotics activity [11]. Valentini et al. found that the cactus pear (L.) Miller), as an alternative substitute feeding, increased the antioxidant capacity of donkey milk [12].

In short, OG has good solubility, high viscosity, thickening, and other processing characteristics, which improve the texture and structure of yogurt, and it also has prebiotic properties. Therefore, OG has a good application prospect in the production of functional yogurt. However, it is necessary to study the effects of OG on the rheological properties, microstructure, and sensory evaluation of set-type yogurt.

Compared with traditional rheology, micro-rheology can analyze the microstructure of soft matter in the static state, and also be used to study the food gel process, without applying an external force to destroy the gel structure, nor affecting the original state of the sample [13,14]. In this work, the quality characteristics of set-type yogurt were evaluated by water-holding capacity (WHC), sensory evaluation, and rheological characteristics, and the suitable concentration of OG was selected. Meanwhile, the acidity of set-type yogurt samples was measured, and the microstructure was observed by scanning electron microscope (SEM). This study will provide a theoretical basis for the application of OG in set-type yogurt.

## 2. Results

### 2.1. WHC

**Figure 1:**
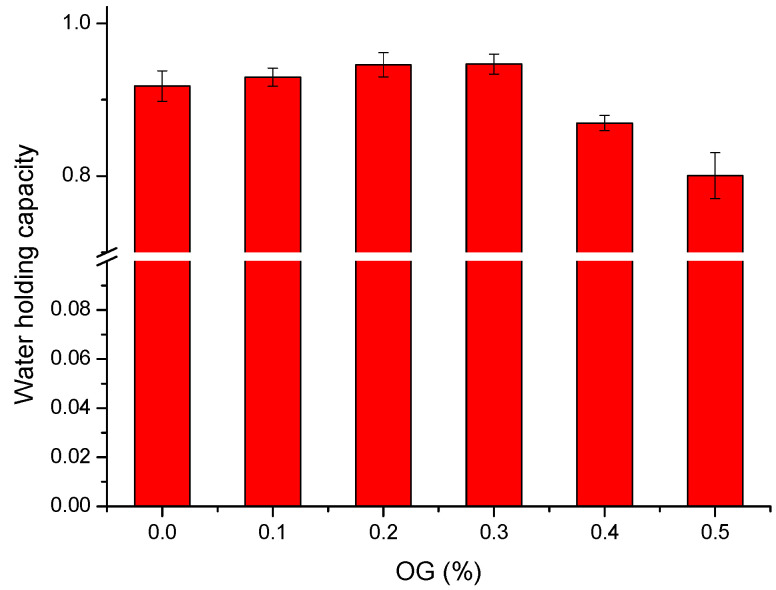
Comparison of WHC of set-type yogurt with different OG concentrations (0, 0.1%, 0.2%, 0.3%, 0.4%, and 0.5%).

As can be seen from Figure 1, when the OG concentration increased from 0 to 0.3%, the WHC gradually increased. At 0.3% OG, the set-type yogurt had the highest WHC of 94.67%. Additionally, the WHC continuously decreased, reaching the lowest WHC (about 80%) at 0.5% OG.

### 2.2. Sensory Evaluation

The sensory evaluation result of set-type yogurt is shown in Table 1. When the OG concentration increased from 0 to 0.3%, the sensory scores gradually increased. At 0.3% OG concentration, the set-type yogurt had the highest sensory score (about 85). Additionally, then, the sensory scores continuously decreased, reaching the lowest sensory scores (about 82) at 0.5% OG concentration. That was in agreement with the result of WHC.

### 2.3. Rheological Characteristics

**Figure 2:**
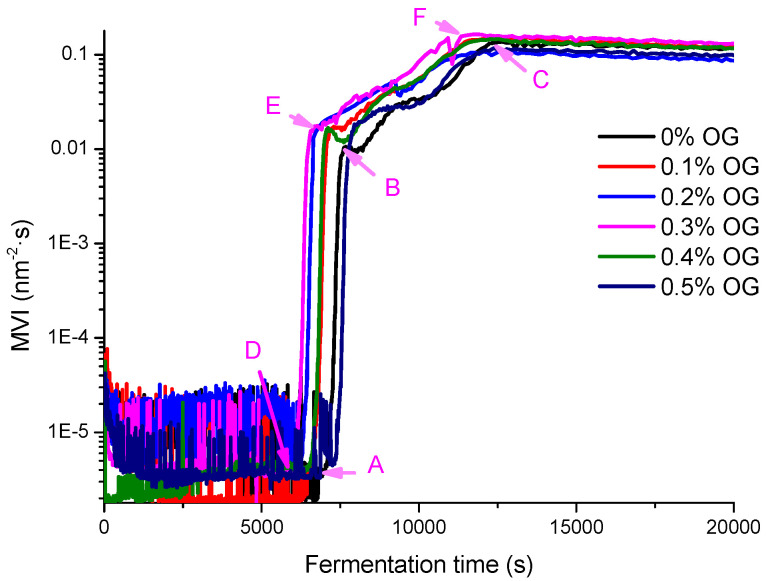
The MVI value curve of set-type yogurt with different OG concentrations (0%, 0.1%, 0.2%, 0.3%, 0.4%, and 0.5%) during fermentation.

**Figure 3:**
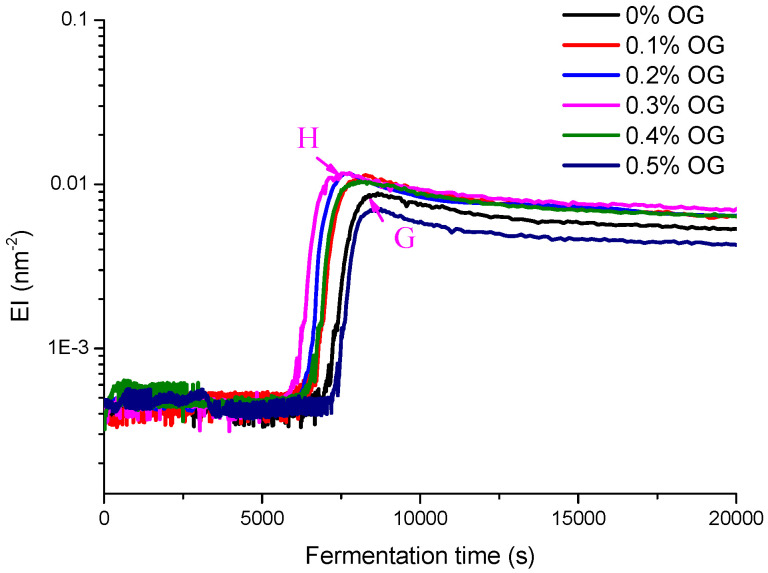
The EI value curve of set-type yogurt with different OG concentrations (0%, 0.1%, 0.2%, 0.3%, 0.4%, and 0.5%) during fermentation.

**Figure 4:**
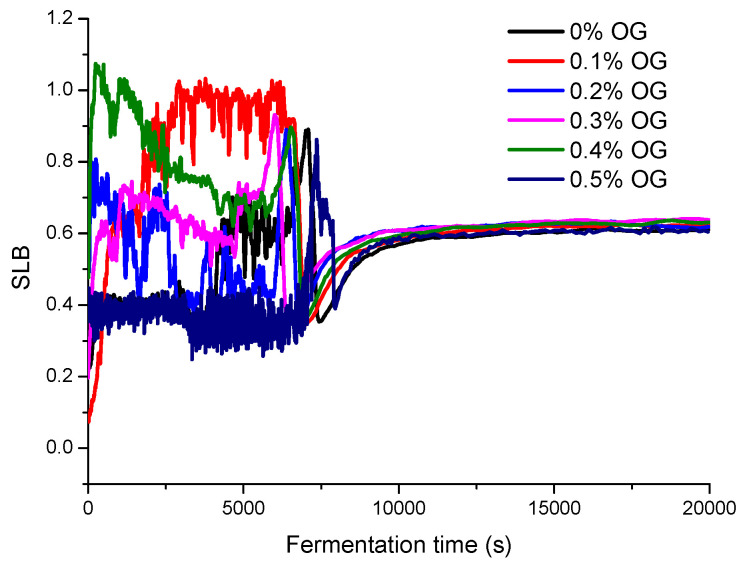
The SLB value curve of set-type yogurt with different OG concentrations (0%, 0.1%, 0.2%, 0.3%, 0.4%, and 0.5%) during fermentation.

In this section, diffusing wave spectroscopy (DWS) method was used to monitor the macroscopic viscosity index (MVI), elasticity index (EI), and solid–liquid balance (SLB) values of set-type yogurt with and without the OG during the fermentation and post-ripening (Figure 2, Figure 3 and Figure 4).

The results showed that the viscosity change of yogurt with OG was similar to that of yogurt without OG. After the stable system was formed, the viscosity of yogurt with OG was higher than that of yogurt without OG. According to the changes of EI values and SLB values, the addition of 0.3% OG increased the liquid behavior of yogurt and shortened the gel point.

According to WHC, sensory evaluation, and rheological characteristics results, the optimal addition amount of OG is 0.3%.

### 2.4. Acidity and pH

**Figure 5:**
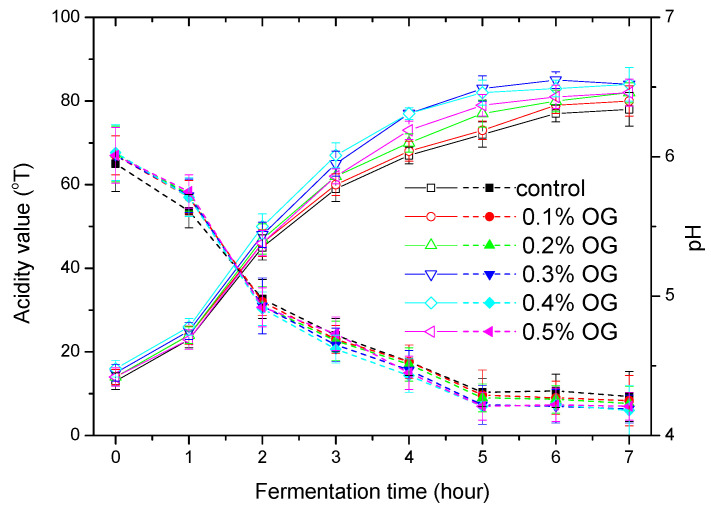
Changes of titrated acidity and pH during fermentation of set-type yogurt with and without OG addition.

As can be seen from Figure 5, the acidity of the set-type yogurt samples increased with the fermentation time. When the OG concentration increased from 0 to 0.4%, the acidity of set-type yogurt gradually increased. At OG concentrations of 0.3% and 0.4%, the set-type yogurt had the highest acidity (about 84). However, at 0.5% OG concentration, the acidity decreased to 82, equal to that at OG concentration of 0.2%. An opposite trend was observed for pH values and reached the minimum (about 4.18) at 0.3% and 0.4% OG. All pH values ranged from 4.18 to 4.28, which are within the normal ranges for set-type yogurts.

### 2.5. SEM

**Figure 6:**
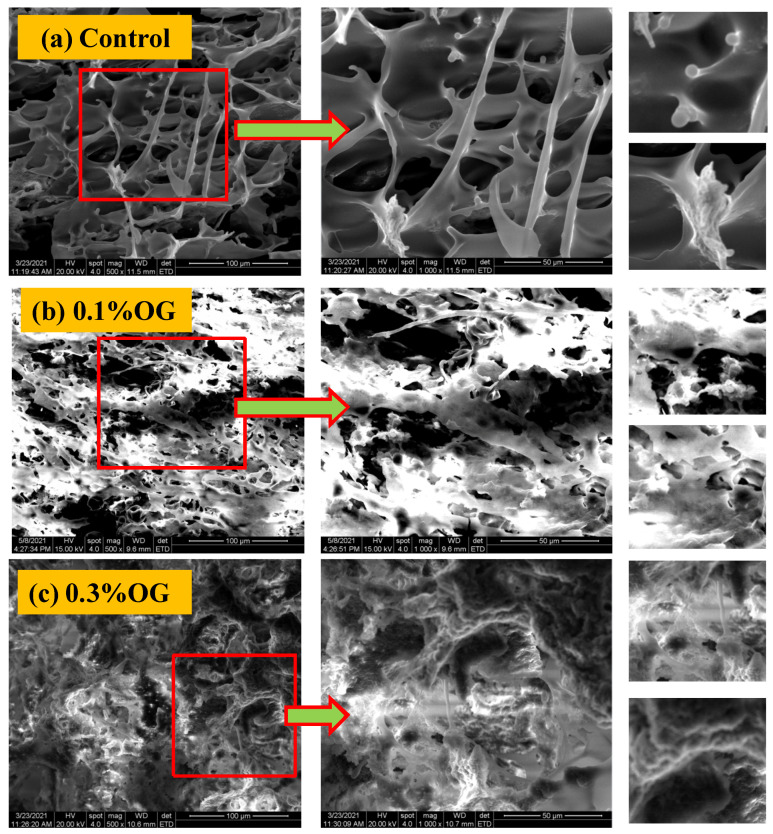
SEM of set-type yogurt before and after adding OG: (**a**) SEM of set-type yogurt without OG addition; (**b**) SEM of set-type yogurt with 0.1% OG; (**c**) SEM of set-type yogurt with 0.3% OG.

As shown in Figure 6a, a clear three-dimensional network is observed for set-type yogurt without OG. When the OG was added, the structures of yogurt changed significantly, depending on the OG concentration. In the set-type yogurt with 0.1% OG, the three-dimensional network of casein was partly destroyed (Figure 6b), while in the set-type yogurt with 0.3% OG, most of the three-dimensional network of casein was destroyed, and some spherical aggregate particles could be clearly observed (Figure 6c).

## 3. Discussion

### 3.1. WHC Analysis

WHC can affect the taste and tissue of set-type yogurt. The better the WHC of yogurt is, the more bound water there is in its gel structure, and the better the taste is [15,16]. The results showed that adding a proper amount of OG could enhance the water stability of set-type yogurt. The reason may be that the OG has a strong water-holding ability, and the interaction between OG and casein can effectively intercept water, prevent the precipitation of whey, and enhance the gel structure of the set-type yogurt system [17]. However, excessive OG might hinder the interactions of OG with casein, destroyed the formation of casein–OG mesh structure, and reduced the WHC of yogurt.

### 3.2. Sensory Evaluation Analysis

The results of Table 1 indicated that the color and flavor of OG yogurt were significantly different from those without OG (*p* < 0.05), but the texture of yogurt was not significantly different (*p* > 0.05) when the amount of OG was less than or equal to 0.4%. The comprehensive score showed that the sensory evaluation of yogurt was acceptable.

### 3.3. Rheological Characteristics

The preparation process of set-type yogurt included fermentation and post-ripening. During the fermentation stage, the gel structure began to form. In the post-ripening stage, the gel structure was further changed, forming a “mature” yogurt system. Hemar et al. had successfully monitored the processing of fermented milk using the DWS method [18,19].

MVI can directly reflect the viscosity characteristics of the system. It can be seen from Figure 2 that, as a function of fermentation time, the fermentation process of the set-type yogurt is a multistage process. Before fermentation, MVI for the set-type yogurt without OG was 0.4 × 10^−5^ at 117.6 min (point A), which was called the initial stagnation stage with low viscosity. Additionally, the fermentation of the set-type yogurt started and the fermentation process entered the rapid viscosity change stage. The viscosity was increased rapidly. At 128.3 min (point B), MVI was 0.01, 2500 times, compared to that of the MVI before fermentation. This is because fermentation led to a gradual decrease in the pH and an increase in the acidity. At the same time, casein particles aggregated to form a gel network structure, which led to an increase in the viscosity of yogurt [20]. At 215.4 min (point C), the set-type yogurt enters the high viscosity stage. The fermentation process ended and a stable gel system was formed. At this stage, MVI increased to the maximum (point C; 0.13), which was 3250 times and 1.3 times, compared to that of the initial stagnation stage and the high viscosity stage, separately.

For the set-type yogurt with OG, although the viscosity change was similar to that of the set-type yogurt without OG, the fermentation time was changed significantly. As shown in Figure 2, when the OG concentration increased from 0 to 0.3%, the fermentation time gradually shortened. At 0.3% OG concentration, the fermentation start time and end time were shortened by 15.1 min (point D; 102.5 min) and 12 min (point F; 203.4 min), respectively. In addition, at point F, the MVI is 0.025 higher than that of point C, indicating that the OG increased the viscosity of the set-type yogurt.

As a function of time, EI can directly reflect the elastic characteristics of the set-type yogurt. As can be seen from Figure 3, when the set-type yogurt was in the initial stagnation stage, the EI value remained unchanged. As the fermentation process entered the rapid viscosity change stage, the EI value was increased. When the fermentation time was 145 min (G point), the yogurt system without adding OG had the maximum EI value of 0.0085. When the fermentation time was 129 min (H point), and 0.3% OG was added, the EI value reached the maximum value (0.0117). It is generally believed that the gelation rate of yogurt is mainly affected by the temperature and the lactic acid bacteria type. However, the gel point of the set-type yogurt with 0.3% OG was shortened by 16 min, compared with that of without OG. Thus, as a functional food ingredient, OG enhanced the nutritional and functional properties of set-type yogurt and shortened the fermentation time, thus increasing the production efficiency.

The value of SLB directly reflects the solid or liquid properties of the product [21]. SLB value is between 0 and 0.5, indicating that the system tends to be solid. A range of 0.5 to 1.0 indicates that the system tends to be liquid. As can be seen from Figure 4, as a function of fermentation time, the SLB value of the sample without OG was increased rapidly from 0.4 to 0.86 (118 min) and then decreased rapidly to 0.34 (124 min). At 180 min, the SLB value stabilized around 0.60, indicating that the set-type yogurt was stable. According to the change of SLB value, the yogurt fermentation process went through the change process of solid → liquid → solid → liquid. After the fermentation, the system was more inclined to be liquid. Interestingly, the SLB value of the sample with 0.3% OG yogurt was 0.63, which was 0.03 higher than that of yogurt without OG, indicating that 0.3% OG addition increased the liquid behavior of the set-type yogurt.

### 3.4. Acidity and pH Analysis

The acidity of set-type yogurt with OG was slightly higher than that of without OG, and the pH was slightly lower. This was because OG, as a prebiotic, can help the *Lactobacillus* to produce acid during the fermentation process [11,21].

### 3.5. SEM Analysis

In general, the three-dimensional network structure of yogurt is considered to be formed by the aggregation of casein. As shown in Figure 6a, the casein aggregates were clearly seen in the set-type yogurt without OG. No lactic acid bacteria were observed (Figure 6a). The reason is possible that the lactic acid bacteria were damaged during vacuum freeze-drying, which was used in the sample pretreatment process. However, there were some high-brightness aggregates that adhered to the casein network. We hypothesized that these aggregates were the whey and exopolysaccharides [22].

The addition of 0.1% OG partly destroyed the three-dimensional network of casein in the set-type yogurt, suggesting that OG can affect the structure of yogurt by its interaction with casein. When the OG concentration was 0.3%, the interaction of OG and casein in the set-type yogurt was more obvious, which is supported by the observation of the spherical structures. These structures were similar to the microstructures of the complex formed by OG and lactoferrin, previously reported by Yang et al. [20].

We hypothesize that due to the addition of OG, the interaction between casein particles was disturbed, and therefore, the three-dimensional aggregates cannot form during fermentation. We hypothesize that they are the aggregates formed by casein particles aggregating on the OG molecular chain during the fermentation process of yogurt. OG is an unbranched flexible polysaccharide polymer, consisting of linear chains of β-d-glucopyranosyl units [21]. Some researchers reported that interactions exist between OG and proteins, such as lactoferrin, soy protein isolate, gliadin, and whey protein [20,23,24]. Therefore, there are some protein-binding sites on its sugar chain. When OG is added to yogurt, casein can attach to the corresponding sites of the OG chain and form protein clusters. As yogurt ferments, these casein proteins are able to aggregate further, forming spherical structures.

## 4. Materials and Methods

### 4.1. Materials and Starters

Pure milk was purchased from Yili Industrial Group Co. Ltd. (Baotou city, Neimenggu, China). Oat β-glucan (95% purity) was purchased from Zhongkang Food Co., (Guangzhou, China). Starters: *Streptococcus thermophilus* and *Lactobacillus bulgaricus (Lactobacillus dechellii Bulgarian subspecies)* (viable bacteria count was about 1 × 10^9^ CFU/g) were purchased from Danisco (China) Co., Ltd., (Shanghai, China). All other chemicals were used of analytical grade.

### 4.2. Methods

#### 4.2.1. Sample Preparation

The various amounts of OG (0.1%, 0.2%, 0.3%, 0.4% and 0.5%; *w*/*w*., %) was added to pure milk, respectively. After stirring, the milk was sterilized at 95 °C for 5 min and then cooled to 43 °C, added the starters (containing *Streptococcus thermophilus* and *Lactobacillus denderi Bulgarian subspecies*), fermented at 43 °C for 5 h, and stored at 4 °C for 24 h.

#### 4.2.2. Determination of WHC

Weighted the 50 mL blank centrifuge tube and denoted as W_0_. Then, 30 g of yogurt was put into the centrifuge tube and denoted as W_1_. After that, the sample was centrifugated at 4000 r/min, for 25 min at 4 °C, discarded the supernatant and weighted, denoted as W_2_.

The calculation formula is as follows:

(1) $$\mathrm{WHC}\;(\%)=\frac{W_{2}-{\;W}_{0}}{W_{1}-W_{0}}\times 100$$

#### 4.2.3. Sensory Evaluation

According to Table 2, 10 volunteers (5 male and 5 female), who had experience in sensory evaluation of fermented milk, were selected to conduct a sensory evaluation of set-type yogurt with different amounts of OG.

#### 4.2.4. Effect of OG on the Rheological Characteristics of Set-Type Yogurt

According to the methods of Titapiccolo et al. [25] and He et al. [22], the rheological characteristics of set-type yogurt were measured by optical microrheometer (RHEOLASER, Formulaction company, Toulouse, France). The changes of MVI, EI, and SLB with time during the gel process were observed at 42 °C. The micro-rheological data of the system were analyzed by RHEOLASER software’s master optical micro-rheological analyzer.

#### 4.2.5. Determination of Acidity and pH

Determination of acidity by an acidimeter (PB-10, Seidolis instruments, Gottingen, Germany) was as follows: set-type yogurt samples were taken every 1 h during the fermentation process, and yogurt samples were determined after being stored 24 h. Then, 10 g yogurt samples were put into a 250 mL triangle bottle, and 20 mL distilled water was added to dilute and mixed, 0.5% phenolphthalein was added as an indicator and titrated with 0.1 mol/L NaOH standard solution until it was slightly red. Not fading within 30 s marked the end of experiment. Consuming 0.1 M of NaOH is equal to 1 °T.

All pH values were monitored after adding starter bacterial cultures, using a digital pH meter (Thermol Scientific Inc., Waltham, MA, USA). 

#### 4.2.6. Determined the Microstructure

According to the method of Cui bo et al. [26], OG samples stored for 24 h after post-ripening were evenly and thinly coated on the inner wall of the Petri dish. After being frozen in liquid nitrogen, the samples were quickly put into a vacuum freeze dryer for drying treatment, and then the samples were prepared by scanning electron microscope (OXFORD INCA250, Shanghai Oxford Instrument Technology Co., Ltd., Shanghai, China). Finally, the images were observed and collected under 10 kV voltage and 500~1000 times magnification.

#### 4.2.7. Statistical Analysis

All experiments were conducted in triplicate, and the results are expressed as the mean ± standard deviation. SPSS version 17.0 (SPSS Inc., Chicago, IL, USA) was used for all statistical evaluations, and OriginPro 8.6.0 (Originlab, Northampton, MA, USA) was used for the construction of the graphs. The sensory evaluation and yogurt WHC with different amounts of OG were analyzed by independent sample t-test of SPSS 17.0 software. One-way ANOVA was used to analyze the titration acidity of yogurt for different fermentation times, and LSD and Dunnett’s T3 test were applied for multiple comparisons; differences were considered to be statistically significant at *p* < 0.05.

## 5. Conclusions

The OG was successfully added into set-type yogurt as a functional ingredient. The fermentation time, WHC, sensory evaluation, and viscoelasticity of the set-type yogurt changed with increasing OG concentration. At 0.3% OG, the set-type yogurt had the highest WHC of 94.67%, the highest score of sensory evaluation, and the shortest fermentation time. The fermentation process of the set-type yogurt went through the changes as follows: solid → liquid → solid → liquid, and the addition of OG made yogurt more inclined to be liquid. The acidity of set-type yogurt with OG was slightly higher. The addition of OG destroyed the three-dimensional network structure of yogurt. In summary, the study provided a detailed characterization of the set-type yogurt with OG.

## Figures and Tables

**Table 1:** Sensory evaluation of set-type yogurt.

OG Addition (%)	Characteristic Index				
	Color	Structural State	Texture	Flavor	Score
0	8.12 ± 0.24	26.05 ± 0.47	24.28 ± 0.39	23.7 ± 0.90	82.15 ± 0.81
0.1	8.6 ± 0.37 **	25.6 ± 0.83	24.35 ± 0.45	24.7 ± 0.64 *	83.25 ± 0.90 *
0.2	8.65 ± 0.45 **	25.4 ± 0.92 *	24.6 ± 0.66	24.6 ± 0.49 *	83.25 ± 0.81 *
0.3	8.71 ± 0.46 *	26.78 ± 0.63 *	24.97 ± 0.79	24.67 ± 0.47 **	85.12 ± 0.89 **
0.4	8.33 ± 0.37 *	25.56 ± 0.49 *	24.67 ± 0.80	24.67 ± 0.46 **	83.22 ± 0.84 *
0.5	8.15 ± 0.32	25.2 ± 0.98	24.8 ± 0.60 *	24.5 ± 0.67 *	82.65 ± 0.95

All data are expressed as the mean ± standard deviation (*n* = 3). * and ** indicate significance at *p* < 0.05 and *p* < 0.01, respectively.

**Table 2:** Sensory score criteria of set-type yogurt.

Item	Scoring Criteria	Score
Color	Uniform color, milky white or milky yellow (7–10)	10
	Different colors (4–6)	
Structural state	Good coagulation, fine and uniform structure, no whey precipitation (25–30)	30
	Good coagulation, fine and uniform structure, a small amount of whey precipitation (15–24)	
	Poor coagulation, different structure, and serious whey precipitation (0–14)	
Texture	The taste is smooth and delicate, with thickness and viscosity (25–30)	30
	The taste is smooth and delicate, with thickness and viscosity (15–24)	
	The taste is not smooth, delicate, and astringent (0–14)	
Flavor	The unique fermented and milk flavor of yogurt, with a strong flavor (25–30%)	30
	The fermented and milk flavor of yogurt is light, and the flavor is general (15–24)	
	Loss of flavor of fermented milk and abnormal odor (0–14)	

## Data Availability

Not applicable.
